# Chemo-Enzymatic Synthesis of Perfluoroalkyl-Functionalized Dendronized Polymers as Cyto-Compatible Nanocarriers for Drug Delivery Applications

**DOI:** 10.3390/polym8080311

**Published:** 2016-08-18

**Authors:** Badri Parshad, Meena Kumari, Katharina Achazi, Christoph Bӧttcher, Rainer Haag, Sunil K. Sharma

**Affiliations:** 1 Department of Chemistry, University of Delhi, Delhi 110 007, India; badriparshad27@gmail.com (B.P.); jakhar7meena@gmail.com (M.K.); 2 Institut für Chemie und Biochemie, Freie Universität Berlin, Takustraße 3, Berlin 14195, Germany; kachazi@zedat.fu-berlin.de; 3 Forschungszentrum für Elektronenmikroskopie, Institut für Chemie und Biochemie, Freie Universität Berlin, Fabeckstraße 36a, Berlin 14195, Germany; christoph.boettcher@fzem.fu-berlin.de

**Keywords:** fluorinated amphiphilic polymers, self-assembly, nanocarriers, drug encapsulation, enzymatic release

## Abstract

Among amphiphilic polymers with diverse skeletons, fluorinated architectures have attracted significant attention due to their unique property of segregation and self-assembly into discrete supramolecular entities. Herein, we have synthesized amphiphilic copolymers by grafting hydrophobic alkyl/perfluoroalkyl chains and hydrophilic polyglycerol [G2.0] dendrons onto a co-polymer scaffold, which itself was prepared by enzymatic polymerization of poly[ethylene glycol bis(carboxymethyl) ether]diethylester and 2-azidopropan-1,3-diol. The resulting fluorinated polymers and their alkyl chain analogs were then compared in terms of their supramolecular aggregation behavior, solubilization capacity, transport potential, and release profile using curcumin and dexamethasone drugs. The study of the release profile of encapsulated curcumin incubated with/without a hydrolase enzyme *Candida antarctica* lipase (CAL-B) suggested that the drug is better stabilized in perfluoroalkyl chain grafted polymeric nanostructures in the absence of enzyme for up to 12 days as compared to its alkyl chain analogs. Although both the fluorinated as well as non-fluorinated systems showed up to 90% release of curcumin in 12 days when incubated with lipase, a comparatively faster release was observed in the fluorinated polymers. Cell viability of HeLa cells up to 95% in aqueous solution of fluorinated polymers (100 μg/mL) demonstrated their excellent cyto-compatibility.

## 1. Introduction

Targeted drug delivery using nanocarriers, which addresses specific tissues or organs in the human body, is an area of research interest that has gained significant attention in recent years. In particular, research on water soluble amphiphilic polymers has gained considerable momentum due to their inherent self-aggregation ability to form diverse nanostructures with different morphologies like vesicles [1], helices [2], rods [3], films [4] micelles [5], etc. [6] in aqueous media. Among these, polymeric micelles with a characteristic core-shell structure are useful nanocarriers for systemic and controlled delivery of drugs due to their high loading, small size, long circulation times, and passive accumulation in tumor tissues [7,8,9]. The kind of morphology formed, its size, and stability are found to be sensitively dependent on the molecular structure as well as their physico-chemical properties [10]. Thus, the fabrication of defined structures and morphologies has been a persistent challenge in the field of polymer synthesis. Various interactions like hydrogen-bonding, metal–ligand interactions, and hydrophobic interactions, among others, could result in self-aggregation [11,12,13], whereas hydrophobic interactions play a major role in driving the aggregation of polymeric amphiphiles. Proficient architectures could be obtained by controlling the structure of polymer backbone and grafted functional groups [6,14]. Grafting of polymers with suitable functional groups has proved to be a good technique for the improvement of chemical and physical properties of amphiphilic polymers, and for the synthesis of polymers of particular interest. Grafting of base polymers with different alkyl chains and [G1.0]/[G2.0] generation polyglycerol dendrons (PG dendrons) has been studied by our group [15,16]. From our previous work on grafting of PG dendrons, we have shown that [G2.0]PG dendrons enhance the stability as well as the biocompatibility of the resulting nanocarriers in aqueous solutions [15,16,17].

The replacement of the alkyl chain with a perfluorinated chain is known to reduce side chain interactions among them, owing to comparatively denser and less polarizable electron cloud around fluorine atoms in perfluoroalkyl chains compared to those around hydrogen in alkyl analogs [18,19]. However, the larger size of fluorine atoms in perfluoroalkyl chains as compared to hydrogen atoms in alkyl analogs allows them to have a larger surface area, thereby increasing the overall hydrophobic character [20,21]. This requirement of perfluoroalkyl chains to occupy a larger space provides the fluorinated polymers with the unique ability to adopt an all-*trans* helical conformation compared to the zig-zag conformation attained by simple alkyl chains [22]. As a result, the perfluoroalkyl chain grafted polymers would build more rigid and orderly packed structures that allow them to form stable micellar aggregates [20]. Also, they should exhibit a lower critical micelle concentration (CMC) than the corresponding non-fluorinated analogs and thus could be more useful in drug delivery applications [23]. With the aim to develop efficient nanocarriers and based on earlier results, we have synthesized perfluorinated alkyl chain grafted polymers and compared their efficacy to that of simple alkyl chain grafted analogs. We used different ratios of the perfluoroalkyl chain/alkyl chain and PG dendron to optimize the hydrophilic and hydrophobic balance and attaining micellar stability as well as to maximize the encapsulation efficiency of a guest molecule. Also, this study facilitates a comparison of the resulting perfluorinated and non-perfluorinated polymeric architectures in terms of their aggregation behavior and transport potential. The size of the resulting nanostructures formed by synthesized polymers in aqueous solution was studied using dynamic light scattering (DLS) and cryo-TEM measurements. Curcumin was selected as a model drug for studying the transport potential and release profile of synthesized amphiphilic polymers by using UV and fluorescence measurements. A release study of curcumin was performed by selecting one each of the representative fluorinated and non-fluorinated polymers and incubating the guest encapsulated nanocarriers with/without *Candida antarctica* lipase (Novozym 435). Also, dexamethasone, a poorly water soluble fluorine containing steroidal drug, was selected for studying drug solubilization capacity and the transport potential of all synthesized polymers.

## 2. Materials and Methods 

### 2.1. Materials

All the solvents and chemicals used for the synthesis were of analytical grade and were purchased from Spectrochem Pvt. Ltd. (Mumbai, India), SD Fine Chemicals Pvt. Ltd. (Mumbai, India) and Sigma-Aldrich Chemicals (Saint Louis, MO, USA). Novozym 435 (immobilized *Candida antarctica* lipase) was purchased from Novozym A/S, (Bagsvaerd, Denmark). Poly[ethylene glycol bis(carboxymethyl)ether]dimethyl ester and glycerol were dried under a vacuum at 60 °C for 10 h prior to their use. A benzoylated dialysis membrane (*M*_W_ cut off size 1200–2000 Da), purchased from Sigma-Aldrich Chemicals, was used for purification of polymers. All the reactions were monitored using pre-coated TLC plates (Merck silica gel 60 F254, Darmstadt, Germany) and the spots were visualized by using ceric solution stain. Silica gel (100–200 mesh) was used for column chromatography. Fresh Milli-Q water was used for the preparation of samples for encapsulation and physico-chemical characterization experiments.

### 2.2. Instrumentation and Methods

#### 2.2.1. NMR, IR Spectroscopy, and GPC Analysis

The ^1^H, ^2^D HETCOR, and ^13^C NMR spectra were recorded on a Jeol-400 MHz and Jeol-100.5 MHz spectrometers (Tokyo, Japan), respectively, using the solvent residual peak as a reference for calibrating the spectra. Deuterated solvents, e.g., CDCl_3_, CD_3_OD, and D_2_O, were used for recording the NMR spectra, where chemical shift values are taken on δ scale and coupling constant value (*J*) are in Hz. Infrared spectra (IR) of all the polymers were recorded using a Perkin-Elmer 2000 FT-IR spectrometer (Singapore). The molecular weights ${\bar{M}}_{w}$, ${\bar{M}}_{n}$ and Polydispersity Index (PDI) of the resulting polymers were determined using an Agilent GPC instrument (Santa Clara, CA, USA) equipped with Agilent 1100 pump, refractive index detector, and PL gel columns using THF at a flow rate of 1.0 mL/min and molecular weights were calibrated using polystyrene standards or pullulan standards.

#### 2.2.2. Critical Aggregation Concentration (CAC) Measurement

The CAC of the polymers was determined by the fluorescence method, using pyrene as a model dye [24]. A stock solution of pyrene was made by dissolving 0.13 mg of pyrene in 1 mL of acetone to form a 6.4 × 10^−4^ M solution. The pyrene solution (10 μL) was taken into empty vials and the acetone was evaporated completely. The polymer solutions of different concentrations (0.25 mM to 0.24 μΜ) were also prepared up to 2 mL using 1× PBS buffer. These polymer solutions were stirred for almost 1 h and then transferred to the vials having pyrene in the same sequence and allowed to mix overnight. The final concentration of pyrene was kept at approximately 3.2 × 10^−6^ M. All the solutions were filtered using 0.22 μm polytetrafluoroethylene (PTFE) filter to remove the non-encapsulated dye. The fluorescence spectra were recorded for the filtered clear solutions and from the fluorescence intensity data, I_1_ (λ_1_: 373 nm) and I_3_ (λ_3_: 384 nm), the ratio of I_3_/I_1_ was calculated and plotted with the log [polymer concentration] to obtain the CAC values.

#### 2.2.3. Dynamic Light Scattering (DLS) and Cryogenic Transmission Electron Microscopy (cryo-TEM)

Malvern Zetasizer Nano ZS analyzer integrated with 4 mW He–Ne laser, λ = 633 nm, using back scattering detection (scattering angle θ = 173°) with an avalanche photodiode detector, was used for determining the size of nanostructures (micelles/aggregates) formed by the supramolecular organization of amphiphilic polymers in the aqueous solution. The samples were prepared by dissolving polymers at the concentration of 3 mg/mL in Milli-Q water and then further allowed to mix at 25 °C for 20 h with 600 rpm. The obtained solutions were then filtered through 0.22 μm PTFE filters, transferred to UV-transparent disposable cuvettes, and used for DLS measurements. To gain further insight into the morphology of nanostructures formed by perfluorinated polymers, their cryo-TEM images were recorded using Tecnai F20 transmission electron microscope (FEI Company, Hillsboro, OR, USA) operated at 160 kV accelerating voltage. The samples for cryo-TEM were prepared by applying the droplets of the sample solution to a 1 µm hole diameter perforated carbon film covering 200 mesh grids (R1/4 batch of the Quantifoil Micro Tools GmbH, Jena, Germany), which had been hydrophilized before use. The extra supernatant fluid was removed using a filter paper to create an ultra-thin layer of the sample solution spanning the holes of the carbon film. The samples were immediately vitrified (to allow artifact-free thermal fixation of the aqueous solution) by propelling the grids into liquid ethane at its freezing point (90 K) with a guillotine-like plunging device. The vitrified samples were then transferred to the microscope using a Gatan (Gatan, Inc., Pleasanton, CA, USA) cryoholder and stage (model 626). The samples were kept at a temperature of 94 K. Imaging was performed using the low-dose protocol of the microscope at a calibrated primary magnification of 62,000× with the defocus set to 1.8 μm. Data were recorded by an Eagle 4k CCD-camera (FEI Company, Hillsboro, OR, USA) set to binning factor 2.

#### 2.2.4. Procedure for Curcumin Encapsulation

The encapsulation of curcumin was monitored by means of a UV–vis spectrophotometer using a Cary-300 instrument from Agilent Technologies and a fluorescence spectrometer using Cary Eclipse. The encapsulation of curcumin was followed by the mixed dispersion method [25]. For encapsulation study, 1 mg of curcumin was weighed and dissolved in 2 mL of acetone. In another vial, polymer solutions of 3 mg/mL concentration were prepared by dissolving in 1× PBS buffer. The prepared curcumin solution in acetone was then added drop wise to the vial having an aqueous polymeric solution. The resulting dispersion was further allowed to mix with the lid open for 24 h by covering the vials with aluminum foil. After 24 h, if the smell of acetone persisted, the traces were removed under reduced pressure on the rotary evaporator. The resulting dispersions were filtered using 0.22 μm PTFE filter and characterized by UV–vis spectrophotometer (200–800 nm) and fluorescence spectrometer (450–800 nm) for absorbance and fluorescence intensity (emission spectra) measurements respectively. The quantification of curcumin was made by lyophilizing the known amount of encapsulated sample and then re-dissolving it in methanol for absorbance and emission measurements. Beer–Lambert′s law was applied for calculating the amount of encapsulated curcumin by using a molar extinction co-efficient (ε) of 55,000 M^−1^·cm^−1^ at 425 nm [26].

#### 2.2.5. Enzyme-Triggered Release Study

For the enzymatic release study, the curcumin was encapsulated in polymeric solutions in 1× PBS buffer by using the same procedure used for the quantitative study. After encapsulation, the excess of curcumin was removed by filtration through 0.22 μm PTFE filter followed by addition of a few drops of *n*-butanol and 200 wt % of the enzyme. The final solutions having pH 7.4 were incubated with 600 rpm at 37 °C for 12 days under dark conditions. Fluorescence spectroscopy was used to study the kinetics of time dependent release by measuring the emission maxima. 

#### 2.2.6. Procedure for Dexamethasone Encapsulation

The dexamethasone encapsulation was studied by using the film method in D_2_O. Dexamethasone (50 wt % of the polymer) taken in a vial was dissolved in acetone and the solvent was allowed to evaporate so as to have a thin film of it at the bottom of the vial. The polymeric solutions prepared in D_2_O at the concentration of 10 mg/mL were then added to vials having dexamethasone and further stirred for 20 h at 600 rpm. A blank sample (without polymeric nanocarriers) having the same amount of the drug was also prepared following a similar procedure for comparative study as dexamethasone possesses 1% solubility in water. The non-encapsulated drug was removed by filtering through a 0.22-μm PTFE filter two or three times to obtain clear solutions. Two hundred and fifty microliters of the filtered solution were diluted to 1 mL by using acetonitrile and analyzed by HPLC. The quantification of solubilized dexamethasone was carried out using a Shimadzu LC-2010HT HPLC (Kyoto, Japan) system integrated with an internal UV absorption detector (λ = 242 nm). The mobile phase consisted of water:acetonitrile:phosphoric acid (70:30:0.5, *v*/*v*/*v*) and flow rate was set at 1 mL/min. The retention time of dexamethasone was 23.3 min and the total run time was 25 min. The remaining filtered solutions were submitted as such for ^1^H and ^19^F NMR spectroscopy. 

#### 2.2.7. Cytotoxicity Studies

For the cytotoxicity study the perfluoroalkyl chain grafted polymers were dissolved in a PBS buffer and further diluted to at least 1:10 in a cell culture medium (RPMI with 10% FCS). The study was carried out on HeLa cells at two different time intervals, i.e., after 48 and 72 h using real-time cell analysis (RTCA). Doxorubicin was used as the standard drug at a concentration of 1 μM and a cell culture medium with 10% PBS buffer was used as the control. In short, cells were seeded in a 96-well E-plate that was placed in an RTCA SP device (Roche, Mannheim, Germany) for impedance measurement. After 24 h, the polymeric solutions were added at a final concentration of 10 and 100 μg/mL and impedance was measured at least every 15 min for 72 h. The graphs were plotted using end point data using Graph Pad Prism v5.01.

### 2.3. General Procedure for the Synthesis of Polymers ***5a***–***5d***

Polymer **1** (0.44 mmol), 1,1,1,2,2,3,3,4,4,5,5,6,6-tridecafluoro-8-(octaprop-2-ynyloxy)octane (**2**)/1-(prop-2-yn-1-yloxy)octane (**3**), and propargylated [G2.0]PG dendron (**4**) were mixed in a 100-mL flask and dissolved in 60 mL of a 1:1 mixture of anhydrous dichloromethane and dimethylformamide under a nitrogen atmosphere. Then tris(triphenylphosphine)copper(I) bromide (0.011 mmol, 0.025 equivalent (eq.)) and DIPEA (2.74 mmol, 6.24 eq.) were added and the reaction mixture was stirred for 48 h at room temperature. IR was used for monitoring the progress of the reaction. On completion of the reaction, the solvent was removed and the product was washed four or five times with hexane (by sonication), to remove non-polar reactants and residual copper catalyst, tris(triphenylphosphine)copper(I) bromide. The residual reactants and traces of copper catalyst were removed by dialysis using 2000 MWCO dialysis tubing, against chloroform for 48 h (changing the solvent every 8 h), to yield the final purified functionalized polymers, **5a**–**5d**, on concentrating the dialyzed solution using a rotary evaporator.

#### 2.3.1. Polymer **5a** (50% Perfluoro-alkyl Chain and 50% PG Dendron Functionalized) 

Polymer **5a** was synthesized by grafting the base polymer **1** with compounds **2** (0.5 eq.) and **4** (0.5 eq.) in a 1:1 mixture of DCM and DMF, as a viscous oil in 80% yield. IR (Thin film, cm^−1^): 3436, 2916, 1752, 1353, 1458, 1107; ^1^H NMR (400 MHz, CDCl_3_): δ = 7.99–7.30 (br s, 2H, H-1′), 5.23–5.06 (m, 2H, H-2a), 4.89–4.53 (m, 8H, H-1a and H-3a), 4.19–4.04 (m, 12H, H-α, H-α′, H-2′a, H-2′b), 3.90–3.37 (m, 194H, –(OC*H_2_*C*H_2_*)*_n_*_~20_ & H-1′′), 2.35 (m, 2H, H-2′′). ^13^C NMR (100.5 MHz, CDCl_3_): δ = 170.4, 170.0, 169.7, 117.7, 70.9, 70.8, 70.5, 70.0, 68.4, 68.3, 68.2, 64.5, 63.7, 63.2, 62.3, 61.1, 60.8, 58.4, 51.7, 49.5, 31.5, 31.3, 31.1, 29.6, 29.3, 22.6, 14.1; ${\bar{M}}_{n}$ (NMR analysis) = 14,690 g/mol; GPC (THF, 1 mL/min): ${\bar{M}}_{w}$ = 5552 g/mol, ${\bar{M}}_{n}$ = 4127 g/mol, PDI = 1.3 (Figure S7A).

#### 2.3.2. Polymer **5b** (70% Perfluoro-alkyl Chain and 30% PG Dendron Functionalized)

Polymer **5b** was synthesized by grafting the base polymer **1** with compounds **2** (0.7 eq.) and **4** (0.3 eq.) in a 1:1 mixture of DCM and DMF, as a viscous oil in 75% yield. IR (Thin film, cm^−1^): 3435, 2916, 1752, 1352, 1459, 1108; ^1^H NMR (400 MHz, CDCl_3_): δ = 8.06–7.33 (br s, 2H, H-1′), 5.53–5.19 (m, 2H, H-2a), 5.01–4.67 (m, 8H, H-1a and H-3a), 4.42–4.20 (m, 12H, H-α, H-α′, H-2′a, H-2′b), 4.05–3.52 (m, 144H, –(OC*H_2_*C*H_2_*)*_n_*_~20_ & H-1”), 2.54–2.45 (m, 2H, H-2′′). ^13^C NMR (100.5 MHz, CDCl_3_): δ = 170.2, 170.1, 169.8, 169.7, 169.5, 144.6, 122.4, 70.7, 70.6, 70.3, 68.2, 68.0, 63.5, 63.0, 62.3, 62.1, 61.4, 61.3, 61.0, 60.5, 58.1, 51.5, 49.1, 31.3, 31.1, 30.9, 29.3, 13.9; ${\bar{M}}_{n}$ (NMR analysis) = 14,380 g/mol; GPC (THF, 1 mL/min): ${\bar{M}}_{w}$ = 4336 g/mol, ${\bar{M}}_{n}$ = 3284 g/mol, PDI = 1.3 (Figure S7B).

#### 2.3.3. Polymer **5c** (50% Alkyl Chain and 50% PG Dendron Functionalized)

Polymer **5c** was synthesized by grafting the base polymer **1** with compounds **3** (0.5 eq.) and **4** (0.5 eq.) in a 1:1 mixture of DCM and DMF, as a viscous oil in 74% yield. IR (Thin film, cm^−1^): 3435, 2917, 1752, 1459, 1105; ^1^H NMR (400 MHz, CDCl_3_): δ = 7.90–7.33 (br s, 2H, H-1′), 5.13–5.05 (m, 2H, H-2a), 4.60–4.53 (m, 8H, H-1a and H-3a), 4.25–4.10 (m, 12H, H-α, H-α′, H-2′a and H-2′b), 3.76–3.41 (m, 203H, –(OC*H_2_*C*H_2_*)*_n_*_~20_ & H-1”), 1.56–1.51 (m, 2H, H-2′′), 1.28–1.21 (m, 10H, H-3′′-H-7′′), 0.82 (t, 3H, H-8′); ^13^C NMR (100.5 MHz, CDCl_3_): δ = 170.0, 169.9, 169.8, 169.6, 169.4, 71.0, 70.6, 70.4, 70.1,68.5, 68.3, 68.0, 67.9, 63.8, 62.9, 62.2, 57.9, 31.4, 29.2, 29.0, 28.8, 25.9, 22.2, 13.7; ${\bar{M}}_{n}$ (NMR analysis) = 13,638 g/mol; GPC (THF, 1 mL/min): ${\bar{M}}_{w}$ = 2162 g/mol, ${\bar{M}}_{n}$ = 1431 g/mol, PDI = 1.5 (Figure S7C).

#### 2.3.4. Polymer **5d** (70% Alkyl Chain and 30% PG Dendron Functionalized)

Polymer **5d** was synthesized by grafting the base polymer **1** with compounds **3** (0.7 eq.) and **4** (0.3 eq.) in a 1:1 mixture of DCM and DMF, as a viscous oil in 79% yield. IR (Thin film, cm^−1^): 3435, 2918, 1752, 1459, 1107; ^1^H NMR (400 MHz, CDCl_3_): δ = 7.93–7.35 (br, 2H, H-1′), 5.11–5.06 (m, 2H, H-2a), 4.61–4.52 (m, 8H, H-1a and H-3a), 4.26–4.11 (m, 12H, H-α, H-α′, H-2′a and H-2′b), 3.78–3.42 (m, 147H, –(OC*H_2_*C*H_2_*)*_n_*_~20_ & H-1”), 1.58–1.52 (m, 2H, H-2′′), 1.29–1.23 (m, 10H, H-3′′-H-7′′), 0.83 (t, 3H, H-8′); ^13^C NMR (100.5 MHz, CDCl_3_): δ = 170.1, 169.9, 169.7, 169.6, 169.5, 70.8, 70.6, 70.5, 70.1, 68.6, 68.3, 68.1, 67.9, 63.9, 62.9, 62.3, 57.9, 31.5, 29.3, 29.1, 28.9, 25.8, 22.3, 13.8; ${\bar{M}}_{n}$ (NMR analysis) = 12,907 g/mol; GPC (THF, 1 mL/min): ${\bar{M}}_{w}$ = 2454 g/mol, ${\bar{M}}_{n}$ = 2011 g/mol, PDI = 1.2 (Figure S7D).

## 3. Results and Discussion

The major focus of this work was to compare the effect of perfluoroalkyl and simple alkyl chains on the supramolecular self-assembly behavior of nanocarriers and their transport potential for curcumin and the fluorinated drug, dexamethasone. For this purpose, the amphiphilic graft copolymers were synthesized by grafting the block copolymer of poly[ethylene glycol bis(carboxymethyl) ether]diethyl ester and 2-azidopropan-1,3-diol with perfluoroalkyl chain and the corresponding simple alkyl analog besides the [G2.0]PG dendron using a “click chemistry” approach.

### 3.1. Synthesis and Characterization

**Scheme 1:**
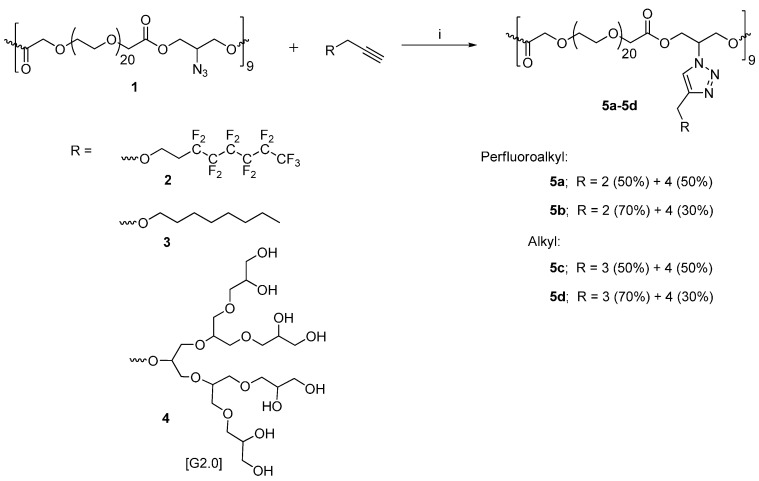
Synthesis of functionalized amphiphilic polymers: (i) [Cu(PPh_3_)_3_]Br, DIPEA, DCM/DMF.

The base polymer was functionalized with varying ratios of 1,1,1,2,2,3,3,4,4,5,5,6,6-tridecafluoro-8-(octaprop-2-ynyloxy)octane (**2**)/1-(prop-2-yn-1-yloxy)octane (**3**) and propargylated [G2.0]PG dendron (**4**) using “click chemistry” to yield the grafted polymers **5a**–**5d** (Scheme 1). 1,1,1,2,2,3,3,4,4,5,5,6,6-tridecafluoro-8-(octaprop-2-ynyloxy)octane (**2**) in turn was synthesized from 3,3,4,4,5,5,6,6,7,7,8,8,8-tridecafluorooctan-1-ol following the literature [27].

All the polymers grafted with perfluoroalkyl/alkyl chain and PG dendron were characterized by their physical and spectral properties. The attachment of a perfluoroalkyl chain was confirmed by the observance of peaks in the ^1^H NMR spectra at around δ 2.35 and 2.49 ppm for polymers **5a** and **5b**, respectively (Figures S1 and S2). Also, the presence of a peak in the aromatic region at around δ 7.30–8.00 ppm in ^1^H NMR confirmed the triazolyl ring proton with its corresponding carbon appearing around δ 120 ppm in the ^13^C NMR spectrum. The peaks around δ 68.2, 68.0 ppm and δ 63.5, 62.1, 61.0 ppm in ^13^C NMR spectrum of polymer **5b** were assigned to C-α, C-α′, C-2′a, C-2′b and C-1a, C-3a respectively, and the methine carbon (C-2a) of polymer **5b** attached to triazole moiety was observed at δ 58.1 ppm. The peak assignment was carried by comparing their cross-peaks in ^2^D HETCOR NMR spectrum; the above assignment was further supported by the DEPT-135 NMR spectrum (Figure S3). The attachment of an alkyl chain in polymer **5c** was confirmed by the observance of a peak at δ 0.82 ppm for the terminal –CH_3_ in ^1^H NMR spectrum, which is further supported by the peaks at δ 1.28–1.21 and 1.56–1.51 ppm for the remaining alkyl chain’s protons. The peaks at δ 13.7, 22.2, 25.9, 28.8, 29.0, 29.2, and 31.4 ppm in the ^13^C NMR spectrum also confirmed the attachment of an alkyl chain to the base polymer (Figure S4). The characterization of polymer **5d** was done in a similar manner (Figure S5). The complete functionalization of azide groups of the base polymer was confirmed by the complete disappearance of the azide peak at 2115 cm^−1^ in the IR spectra (Figure S6). 

### 3.2. CAC Calculation Using Fluorescence Measurement

**Figure 1:**
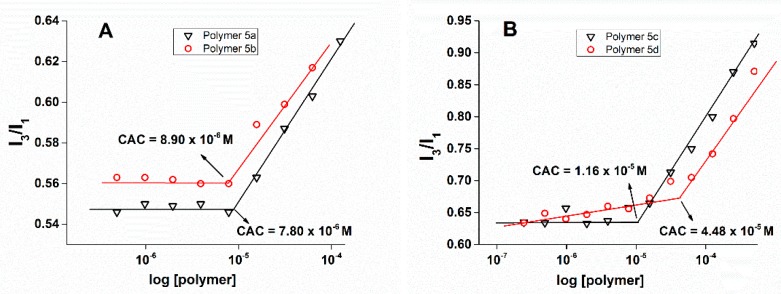
Evaluation of CAC of polymers using pyrene as a hydrophobic probe and by measuring I_3_/I_1_ ratio: (**A**) polymers **5a** and **5b**; (**B**) polymers **5c** and **5d**.

The CAC of the polymers was determined by fluorescence measurements, using pyrene as a model dye. The ratio of I_3_/I_1_ was calculated and plotted with the log [polymer concentration] to obtain the CAC values (Figure 1). The CAC of fluorinated polymers i.e., **5a** and **5b**, was observed to be 8.90 × 10^−6^ M and 7.80 × 10^−6^ M, respectively, less than the CAC of their non-fluorinated analogs (**5c** = 1.16 × 10^−5^ M and **5d** = 4.48 × 10^−5^ M). This trend is in accordance with the literature, wherein the fluorinated amphiphiles displayed lower CAC than their non-fluorinated analogs. The lower CAC values of the polymeric amphiphiles having perfluoroalkyl chains indicate that they form more stable micelles compared to their non-fluorinated analogs. The stability of micelles formed can be explained by the more rigid and orderly packed structure of the perfluoroalkyl chain due to their unique ability to attain an all-*trans* helical conformation [19,22].

### 3.3. DLS and Cryo-TEM Analysis

**Figure 2:**
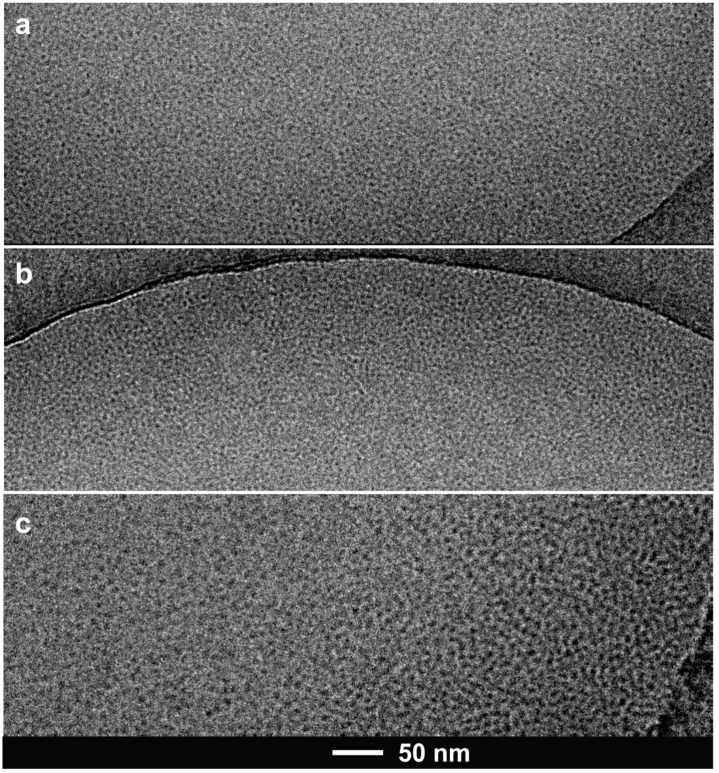
Cryo-TEM images of polymers, (**a**) **5a**; (**b**) **5b** showing individual spherical micelles in the diameter range of 5 nm; (**c**) Cryo-TEM image of individual spherical micelles of ~8 nm in diameter formed upon encapsulation of curcumin encapsulated by polymer **5a**.

The supramolecular self-organization behavior of the synthesized polymers in aqueous solution was studied using DLS measurements at a concentration of 3 mg/mL. The DLS size distribution graphs of polymers **5a**, **5b** and **5c**, **5d** are shown in Figures S8 and S10, respectively. The size distribution profile was found to be bimodal in intensity and monomodal in volume and number for polymers **5a** and **5b**. The presence of two peaks in intensity distribution profile could be assigned to micelles and micellar aggregates, while the appearance of a single peak in volume and number corresponding to the smaller peak in intensity, assigned to micelles, indicated that micelles are the predominant species in the aqueous solution. The obtained data from DLS measurements, summarized in Table 1, indicates that the polymers grafted with perfluorinated alkyl chain form smaller micelles compared to their non-fluorinated analogs. This could be attributed to the additional rigidity provided by highly ordered and dense packing of perfluoroalkyl chains in their core. The zeta potential of the studied polymers (**5a**–**5d**) was also measured and perfluoroalkyled polymers were observed to have a higher negative zeta potential value (−14 to −20 mV) as compared to alkyled polymers (approx. −8 mV) (Figure S17, Table S1). To gain additional insight into the morphology of nanostructures resulting from perfluorinated polymers, cryo-TEM images were taken using 1 mM aqueous solutions. Interestingly, cryo-TEM images of both the polymers **5a** and **5b** showed monodisperse spherical particles with a diameter about 5 nm (Figure 2), thereby supporting the DLS results.

### 3.4. Transport Potential for Curcumin

Curcumin is a hydrophobic drug possessing various therapeutic properties, e.g., antioxidant [28], anticancer [29], and anti-inflammatory [30], among various others. Owing to its potential medicinal value, it is widely used as a dietary supplement in Southeast Asia. Also, it is used in cosmetics, and as a food flavoring and coloring agent [31,32]. However, for medicinal purposes, the actual amount of curcumin that reaches target sites (bioavailability) is very low because of its low aqueous solubility and fast chemical and photochemical degradation. To enhance the bioavailability of curcumin, various approaches have been explored such as drug polymer conjugate formation [33], nanoparticle formation, encapsulation by different nanocarriers [34,35,36], and synthesis of its structural analogs [37]. Among the various approaches studied, the encapsulation strategy seems to be the most suitable due to its target specificity, intact activity, and ease of synthesis.

**Figure 3:**
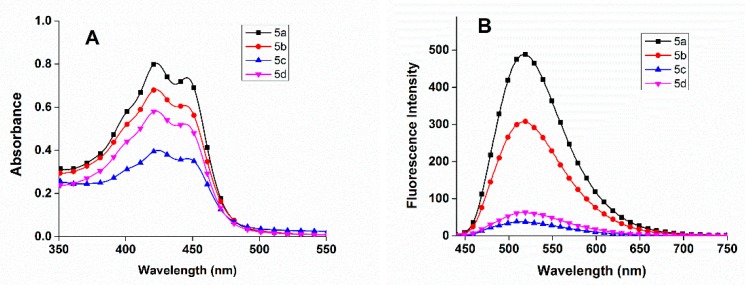
(**A**) Absorbance spectra of curcumin encapsulated samples in methanol; (**B**) emission spectra of curcumin encapsulated samples in methanol.

**Figure 4:**
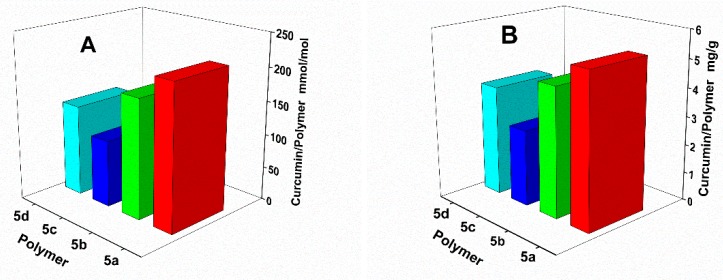
Quantification of curcumin encapsulated by polymers: (**A**) transport capacity; (**B**) transport efficiency.

We have studied the transport potential of polymers by encapsulating curcumin and quantifying its amount from the absorption spectra of encapsulated samples in methanol (Figure 3). Polymer **5a** grafted with 50% perfluoroalkyl chain and 50% PG dendron exhibited the highest encapsulation of curcumin with a transport efficiency of 5.3 mg/g and transport capacity of 213 mmol/mol. Polymer **5b** having 70% perfluoroalkyl chain and 30% PG dendron showed a transport efficiency of 4.5 mg/g and transport capacity of 176 mmol/mol for curcumin encapsulation. However, polymers **5d** and **5c**, grafted with non-fluorinated alkyl chain, were found to have a lower encapsulation potential of 3.9 and 2.7 mg/g (curcumin/polymer), respectively (Figure 4). Hence, the polymers grafted with perfluoroalkyl chain exhibited higher transport potential for curcumin than the corresponding non-fluorinated analogs. This may be due to the presence of a more hydrophobic micellar core in the fluorinated polymers in comparison to their non-fluorinated analogs. A cryo-TEM image was also recorded for the curcumin-encapsulated fluorinated polymer **5a** to gain insight into the resulting morphology (Figure S18). The representative micrograph of the study is shown in Figure 2c and shows homogeneous distribution of spherical particles in the 8 nm range, slightly larger than the unloaded polymer (5 nm, Figure 2a).

### 3.5. Cytotoxicity Study

The perfluoroalkyl chain grafted polymers with high transport potential, i.e., **5a** and **5b** were chosen for the cytotoxicity study on HeLa cells (Figure S11). Both polymers showed nearly no toxicity at test concentrations, 10 μg/mL and 100 μg/mL, after 48 h and 72 h. Thus, the good cell viability values, along with their promising transport potential, qualify these polymers as potential nanocarriers for drug delivery applications.

### 3.6. Enzyme-Triggered Release of Curcumin 

Besides drug encapsulation, triggering its release in a controlled manner to maintain the therapeutic concentration is even more important. Under physiological conditions, enzyme-mediated release of the drug from amphiphilic polymers is of great relevance.

**Figure 5:**
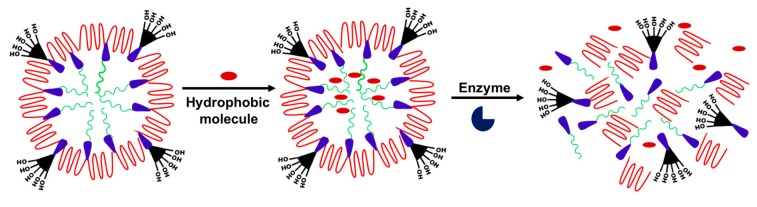
Enzyme-triggered systematic release of hydrophobic molecules from amphiphilic polymers.

**Figure 6:**
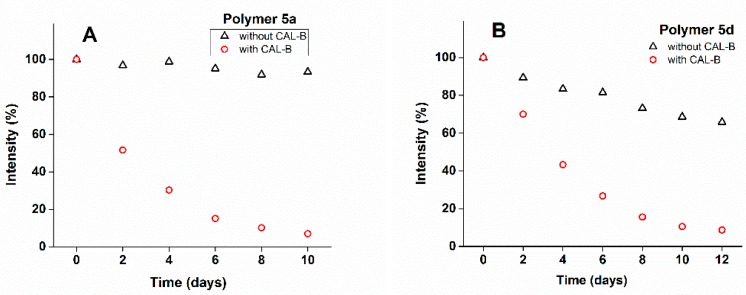
Intensity of emission of curcumin encapsulated in nanocarriers having pH 7.4 (1× PBS buffer) and incubated with and without enzyme at 37 °C (**A**) **5a**; (**B**) **5d**.

Since the polymers reported herein are based on ester linkages and are formed by a lipase mediated *trans*-esterification reaction of PEG-1000 diester and the hydroxyl group of azido glycerol, they may be sensitive to Novozym 435 catalyzed hydrolytic conditions. Thus, the release of encapsulated hydrophobic molecules was studied by incubating the drug-encapsulated nanocarriers with Novozym 435 under dark conditions. A proposed systematic release of the drug from amphiphilic polymers is shown in Figure 5. For comparative release study, Novozym 435 (200 wt %) was added to both the solutions, i.e., curcumin-encapsulated fluorinated polymer **5a** and the non-fluorinated polymer **5d**. The mixture having pH 7.4 was kept at 37 °C and release of curcumin from the nanocarrier was measured for 12 days by comparing the decrease in % fluorescence intensity of the samples in the presence and absence of the enzyme under similar conditions (Figure 6). 

A time-dependent decay of the emission intensity of curcumin was observed for 10–12 days for polymers **5a** and **5d** using fluorescence spectroscopy (Figures S12 and S13). As is evident from Figure 6, the intensity of the yellowish color of curcumin decreased with time in the samples having enzyme as compared to the control without the enzyme. Approximately 50% decay in the intensity occurred within 2–3 days for both the curcumin-encapsulated polymers, and maximum decay (95%–96%) was reached after 10–12 days. In the control experiment without the enzyme, only 6% decay in fluorescence intensity of curcumin was observed for the polymer **5a**, as compared to 34% for **5d**, thus suggesting that the fluorinated polymer **5a** stabilizes the encapsulation of curcumin in its hydrophobic core more efficiently as compared to the non-fluorinated analog **5d**. Comparing the kinetics of enzyme-mediated release of curcumin in the polymeric systems **5a** and **5d**, a faster release was observed in the former (**5a**); this may be due to the smaller size of fluoro polymers as compared to its alkyl analog **5d** [38]. Thus the fluorinated polymers have proved to be better nanocarriers for hydrophobic drugs/dyes in comparison to non-fluorinated analogs. 

### 3.7. Solubilization of Dexamethasone

**Figure 7:**
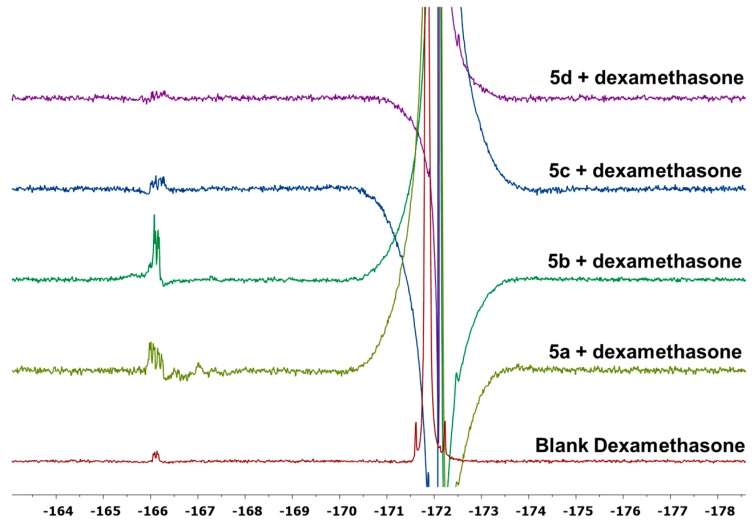
^19^F NMR spectra of dexamethasone encapsulated polymers (**5a**, **5b**, **5c**, and **5d**) and blank dexamethasone in deuterated methanol; 5-fluorouracil (2 mg/mL) used as internal reference.

To investigate the effect of perfluoroalkyl groups in the core of micelles of perfluorinated polymers, dexamethasone, a fluorinated and poorly water soluble drug, was selected for encapsulation purposes. Dexamethasone is a steroidal drug belonging to the glucocorticoid class, which possesses potential anti-inflammatory and immunosuppressant properties [39]. A comparison of the ^1^H and ^19^F NMR spectra of encapsulated samples (perfluorinated polymers + dexamethasone in D_2_O) with control (dexamethasone in D_2_O) confirmed the encapsulation of dexamethasone, whereas the non-perfluorinated polymers exhibit a weak tendency for dexamethasone encapsulation (Figure 7). The comparatively higher encapsulation efficiency of perfluorinated polymers for dexamethasone might be explained by their stable micellar structures resulting from ordered packing of perfluoroalkyl chains in the micellar cores. The DLS measurements of the drug-encapsulated perfluorinated nanocarriers indicated that polymeric micelles do not show a significant increase in size on encapsulation of dexamethasone (Figure S9), suggesting its solubilization in the core of micelles as a result of interactions between the perfluoroalkyl chain and the fluorinated drug. The ^1^H NMR spectra of polymer **5b** (70% perfluoroalkyl chain grafted polymer) with encapsulated dexamethasone and blank dexamethasone (drug in D_2_O, without adding polymeric nanocarrier) are shown in Figure S14. The solutions prepared for ^1^H NMR analysis were lyophilized and re-dissolved in deuterated methanol (having 2 mg/mL of 5-fluorouracil as an internal reference) and analyzed by ^19^F NMR spectroscopy. The ^19^F NMR spectra exhibit peaks for dexamethasone at around 166 ppm and for 5-fluorouracil (internal reference) at about 172 ppm. ^19^F NMR spectra of the dexamethasone encapsulated polymers and blank dexamethasone (control, without polymeric nanocarriers) are shown in Figure 7.

Due to the low solubility of dexamethasone in D_2_O (approx. 1%), a very low intensity peak for it was observed in the blank sample; even its encapsulation in non-perfluorinated polymers (**5c** and **5d**) led to only a slight enhancement in the signal intensity, suggesting a lower encapsulation. However, the observance of the comparatively high intensity peak of dexamethasone on encapsulation in perfluorinated polymers suggests a significant enhancement in its solubilization in the presence of perfluorinated nanocarriers. The amount of dexamethasone encapsulated in perfluorinated polymers was quantified using HPLC. The calibration curve (Figure S15) was prepared using dexamethasone solutions of known concentration to find the amount of drug loaded in polymeric nanocarriers. The HPLC chromatogram of blank dexamethasone and after encapsulation in fluorinated polymeric samples using acetonitrile:water:phosphoric acid (30:70:0.5; *v*/*v*/*v*) shows that the blank (control, without polymeric nanocarriers) solution contains 76 μg/mL, which is very close to its reported solubility data (Figure S16) [40].

However, the perfluorinated polymeric nanocarriers **5a** and **5b** solubilized 106 and 124 μg/mL of dexamethasone, respectively, which is 1.4 and 1.6 times higher than the control. For dexamethasone, the % encapsulation efficiency of the studied polymers **5a** and **5b** was found to be 2.12 and 2.48, respectively. The higher transport potential shown by polymer **5b**, grafted with 70% perfluoroalkyl chain as compared to **5a** having 50% perfluoroalkyl chain grafted, could be explained by the enhanced hydrophobicity resulting from increased perfluoroalkyl chain.

## 4. Conclusions 

Herein, we have successfully compared the amphiphilic perfluoroalkyl-functionalized polymers with their nonfluorinated alkyl analogs in terms of supramolecular aggregation behavior and transport potential. Investigation of the self-assembly behavior of these polymers using DLS and cryo-TEM techniques has shown that perfluoroalkyl-grafted polymers form smaller (8–10 nm) micelles compared to their alkyl chain grafted analogs (100–150 nm). As evidenced by fluorescence and UV–Visible spectroscopy, the fluorinated polymers show better solubilization and good transport potential for the poorly water soluble drugs, viz. curcumin and dexamethasone. Although both types of polymeric systems showed up to 90% release of encapsulated curcumin in the presence of *Candida antarctica* lipase within 12 days, a faster release was observed in fluorinated polymers, which may be due to their better fit to the hydrophobic active site of the enzyme. Also, perfluorinated polymers stabilize curcumin more efficiently, as can be seen by the retention of fluorescence intensity in the absence of enzyme. In addition, the perfluorinated polymers have shown nearly no toxicity in HeLa cells up to a concentration of 100 μg/mL over 72 h. In summary, the good encapsulation potential, low toxicity, and systematic release of a hydrophobic drug under physiological conditions demonstrate the potential of these polymers to act as nanocarriers for the transport of hydrophobic drugs. 

## Figures and Tables

**Table 1:** Critical aggregation concentration (CAC) and particle size distribution profile of the nanostructures in aqueous solution.

Polymer	Hydrophobic unit	Hydrophilic unit	CAC (M)	DLS size (nm)			PDI
	C_8_ chain	PG dendron		Intensity	Volume	Number	
**5a**	50% perfluoroalkyl	50%	8.90 × 10^−6^	8.11	7.05	6.27	0.509
**5b**	70% perfluoroalkyl	30%	7.80 × 10^−6^	10.92	8.33	6.87	0.486
**5c**	50% alkyl	50%	1.16 × 10^−5^	143.5	110.7	83.15	0.291
**5d**	70% alkyl	30%	4.48 × 10^−5^	124.2	96.24	75.94	0.269

## Supplementary Materials

Supplementary Materials can be found at www.mdpi.com/2073-4360/8/8/311/s1.
