# Supplementary material for: Chemo-Enzymatic Synthesis of Perfluoroalkyl-Functionalized Dendronized Polymers as Cyto-Compatible Nanocarriers for Drug Delivery Applications
Source: Polymers (Basel). 2016 Aug 18;8(8):311. doi: 10.3390/polym8080311 (PMC6432502; doi:10.3390/polym8080311)
Supplement: Supplementary file 1 [file polymers-08-00311-s001.pdf]

Supplementary Materials: Chemo-Enzymatic Synthesis of Perfluoroalkyl-Functionalized Dendronized Polymers as Cyto-Compatible Nanocarriers for Drug Delivery Applications

Badri Parshad, Meena Kumari, Katharina Achazi, Christoph Böttcher, Rainer Haag and Sunil K. Sharma

Table of contents

Figure S1. ^{1}H and ^{13}C NMR spectra of polymer **5a**.

Figure S2. ^{1}H and ^{13}C NMR spectra of polymer **5b**.

Figure S3. ^{2}D HETCOR and DEPT NMR spectra of polymer **5b**.

Figure S4. ^{1}H and ^{13}C NMR spectra of polymer **5c**.

Figure S5. ^{1}H and ^{13}C NMR spectra of polymer **5d**.

Figure S6. IR spectra of polymers (**1** and **5a**–**5d**).

Figure S7. GPC chromatogram of polymers **5a**-**5d**.

Figure S8. DLS size distribution graphs of polymers **5a** and **5b**.

Figure S9. DLS size distribution graphs of dexamethasone encapsulated polymers **5a** and **5b**.

Figure S10. DLS size distribution graphs of polymers**5c** and **5d**.

Figure S11. Cytotoxicity study of the polymers **5a** and **5b** at concentration of 10 and 100 µg/mL for 48 and 72 h using HeLa cells.

Figure S12. Fluorescence measurement of curcumin release from polymers **5a** and **5d**, with/without incubation with enzyme.

Figure S13. Time dependent release of curcumin from polymers **5a** and **5d**, with/without incubation with enzyme.

Figure S14. ^{1}H NMR spectra of dexamethasone (Blank), and **5b** + dexamethasone in D_2O.

Figure S15. Calibration graph of dexamethasone. Peak Area: Y-axis and Concentration of dexamethasone: X-axis (in µg/mL).

Figure S16. HPLC chromatogram of dexamethasone encapsulated in fluorinated polymeric samples using acetonitrile:water:phosphoric acid (30:70:0.5, *v*/*v*/*v*) as an eluent.

Figure S17. Mean Zeta potential of Polymers **5a**-**5d**.

Figure S18. Cryo-TEM images of polymers, (**a**) **5a**; (**b**) **5b** (**c**) curcumin encapsulated polymer **5a**.

Table S1. Transport behaviour, Encapsulation efficiency and Zeta Potential of Polymers **5a**-**5d**.

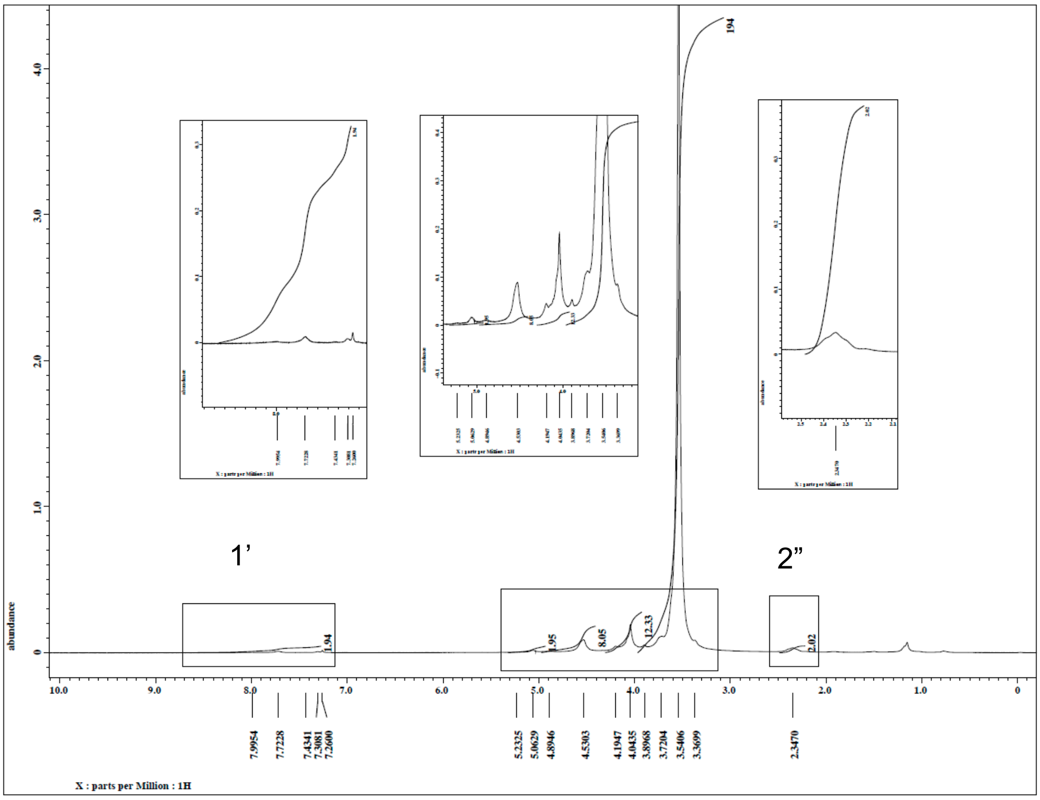

^{1}H NMR spectrum of polymer 5a

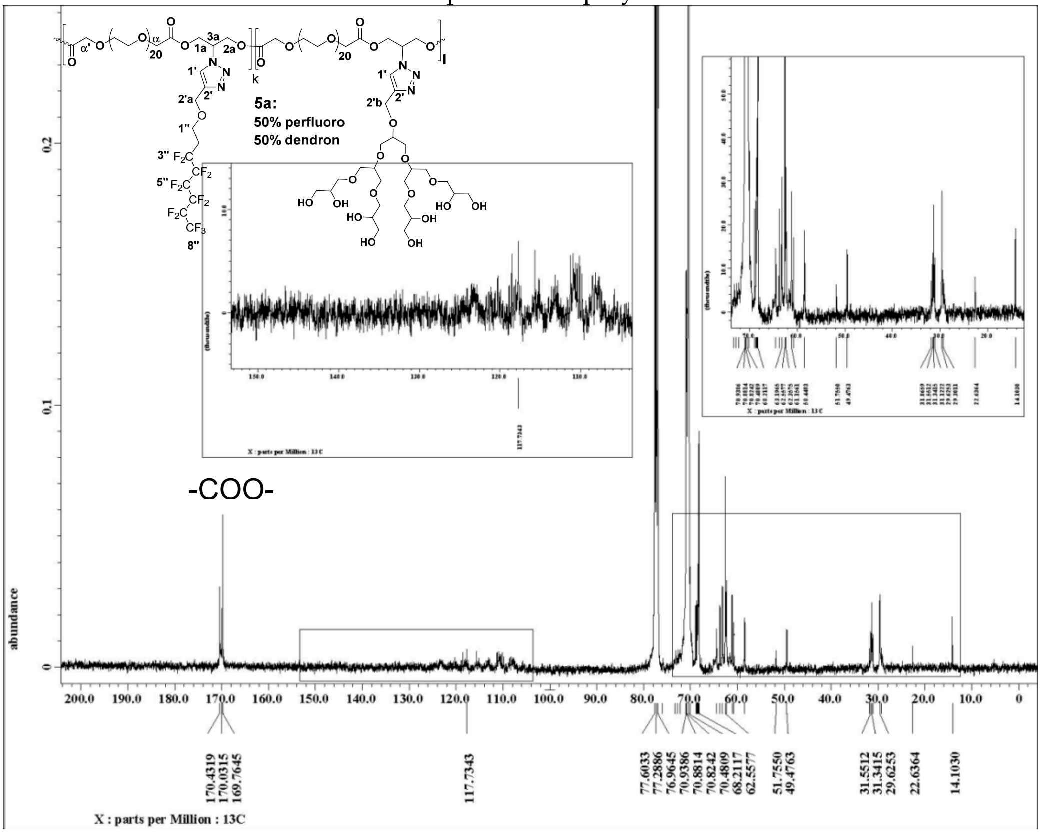

^{13}C NMR spectrum of polymer 5a

Figure S1. ^{1}H and ^{13}C NMR spectra of polymer **5a**.

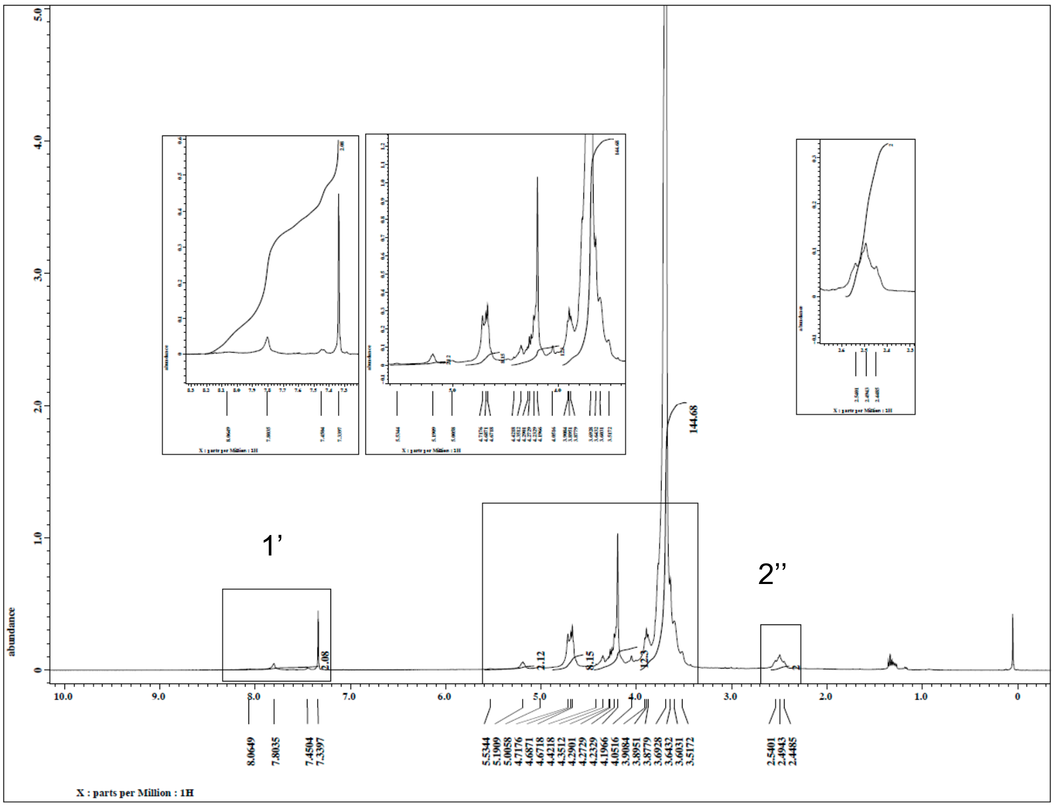

¹H NMR spectrum of polymer 5b

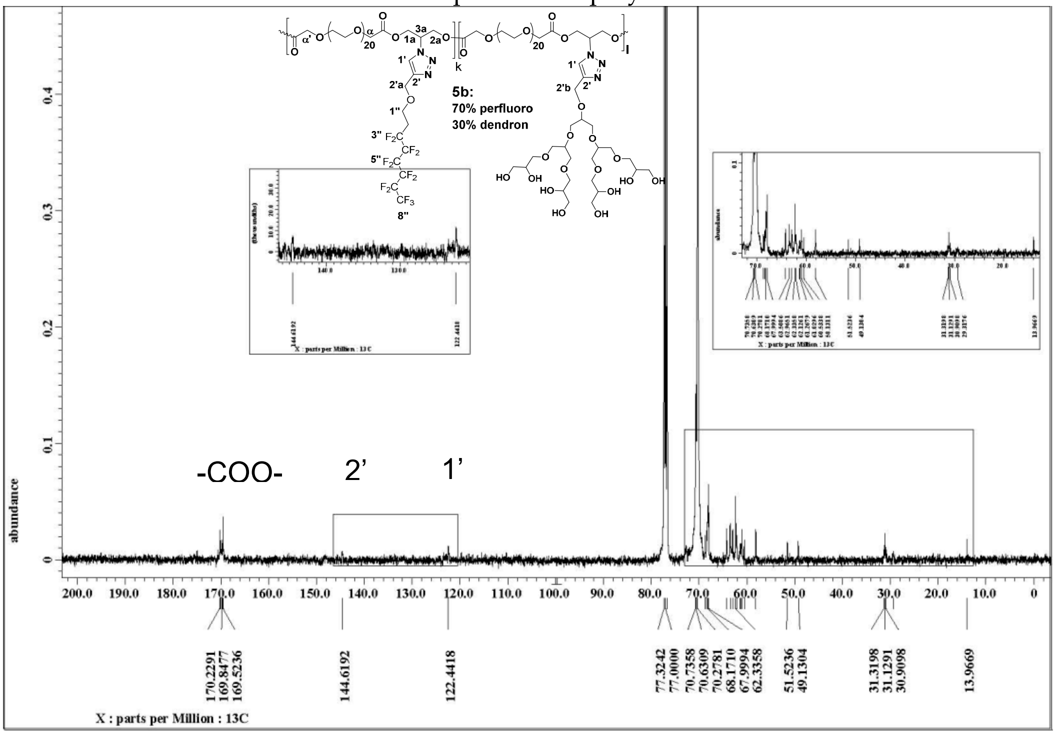

¹³C NMR spectrum of polymer 5b

Figure S2. ¹H and ¹³C NMR spectra of polymer **5b**.

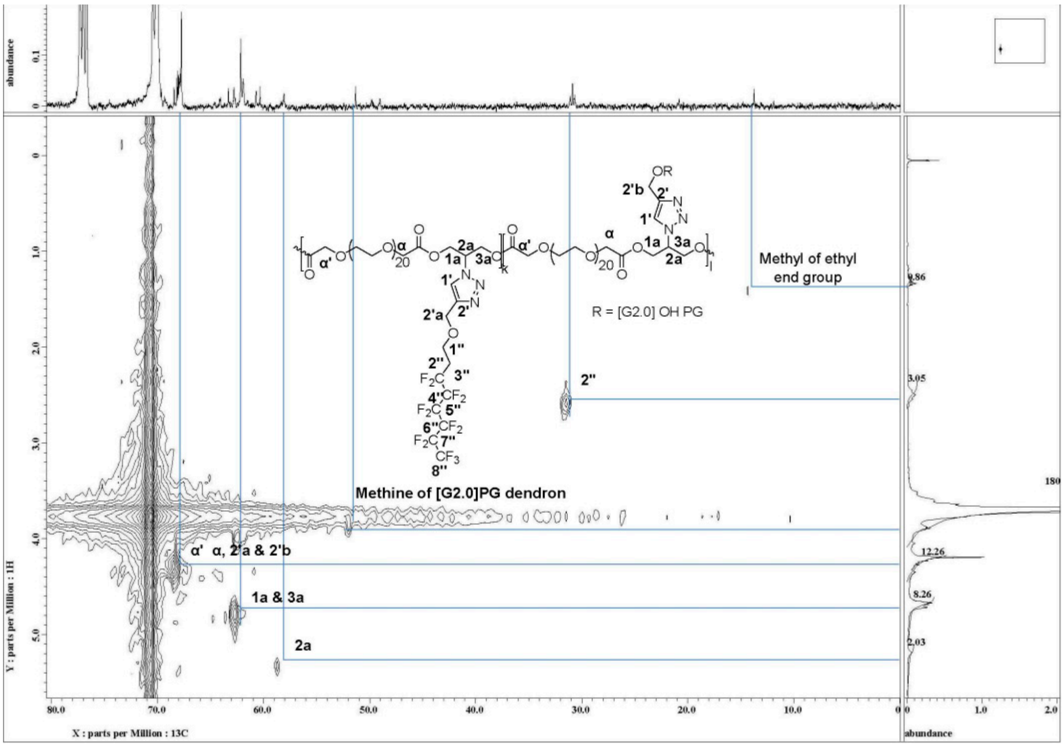

[2]D HETCOR NMR spectrum of polymer 5b

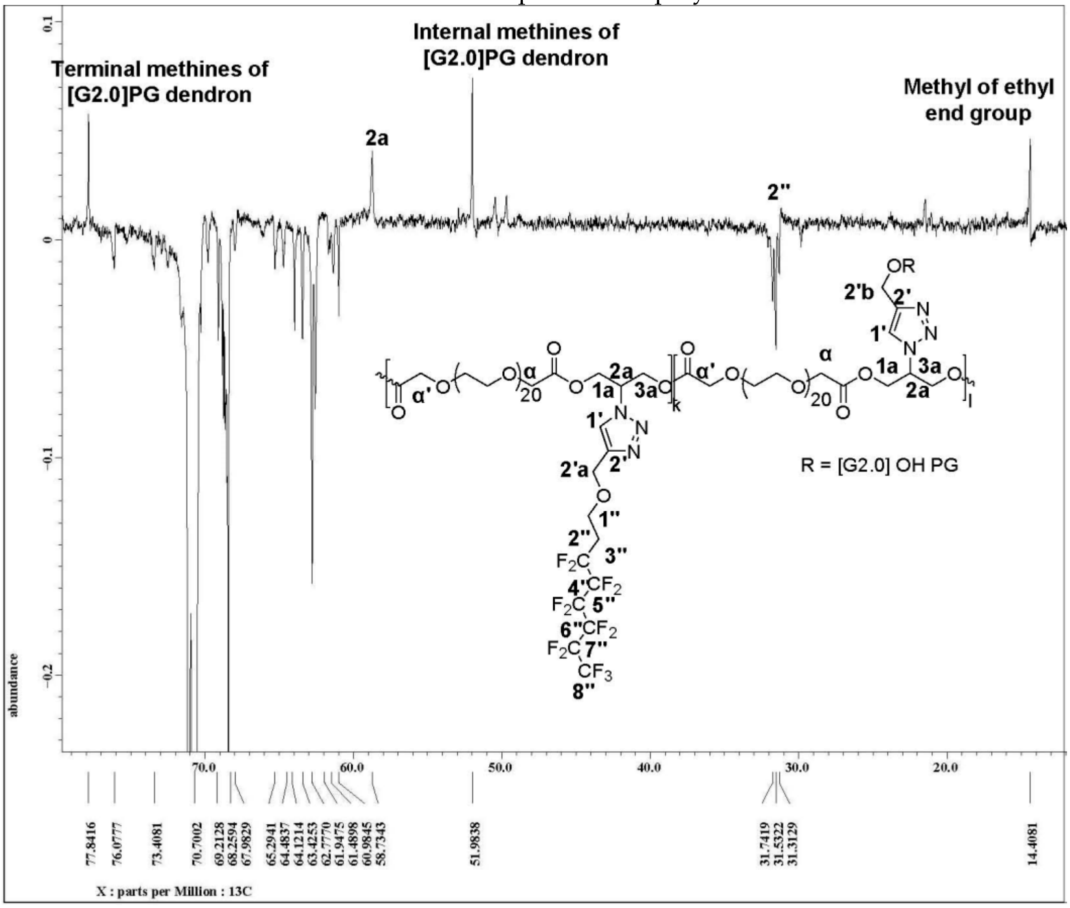

DEPT-135 NMR spectrum of polymer 5b

Figure S3. [2]D HETCOR and DEPT-135 NMR spectra of polymer **5b**.

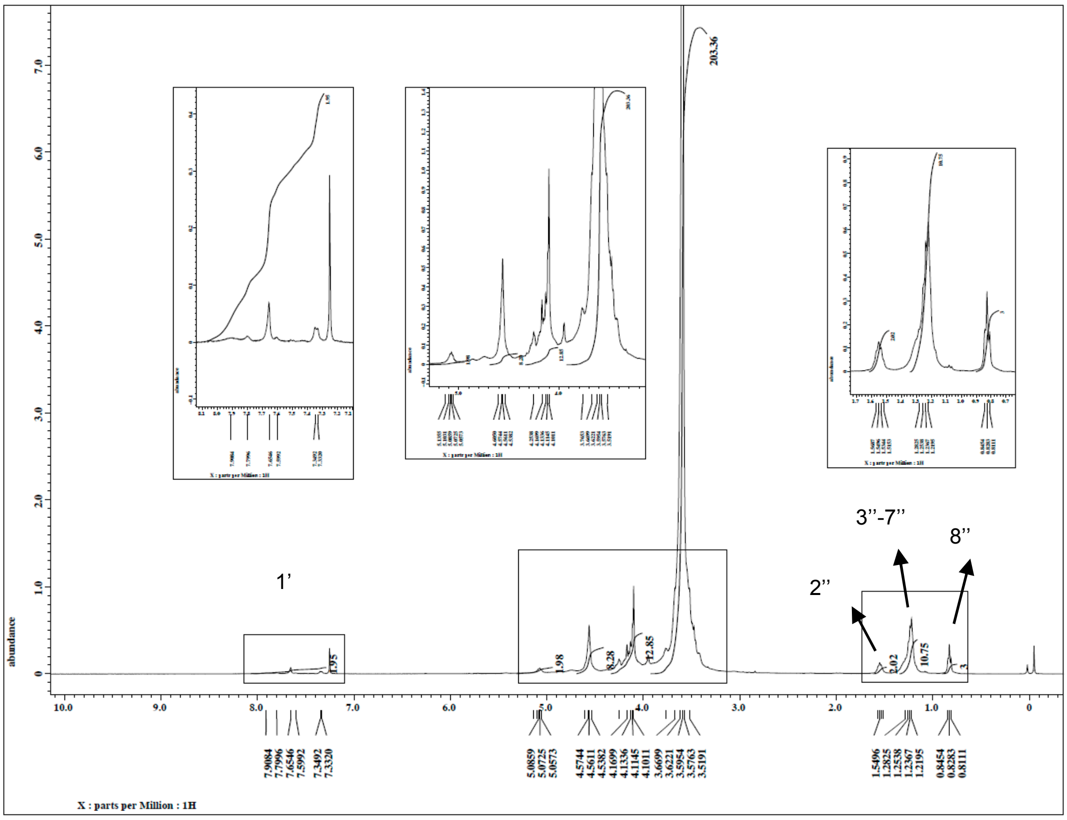

^{1}H NMR spectrum of polymer 5c

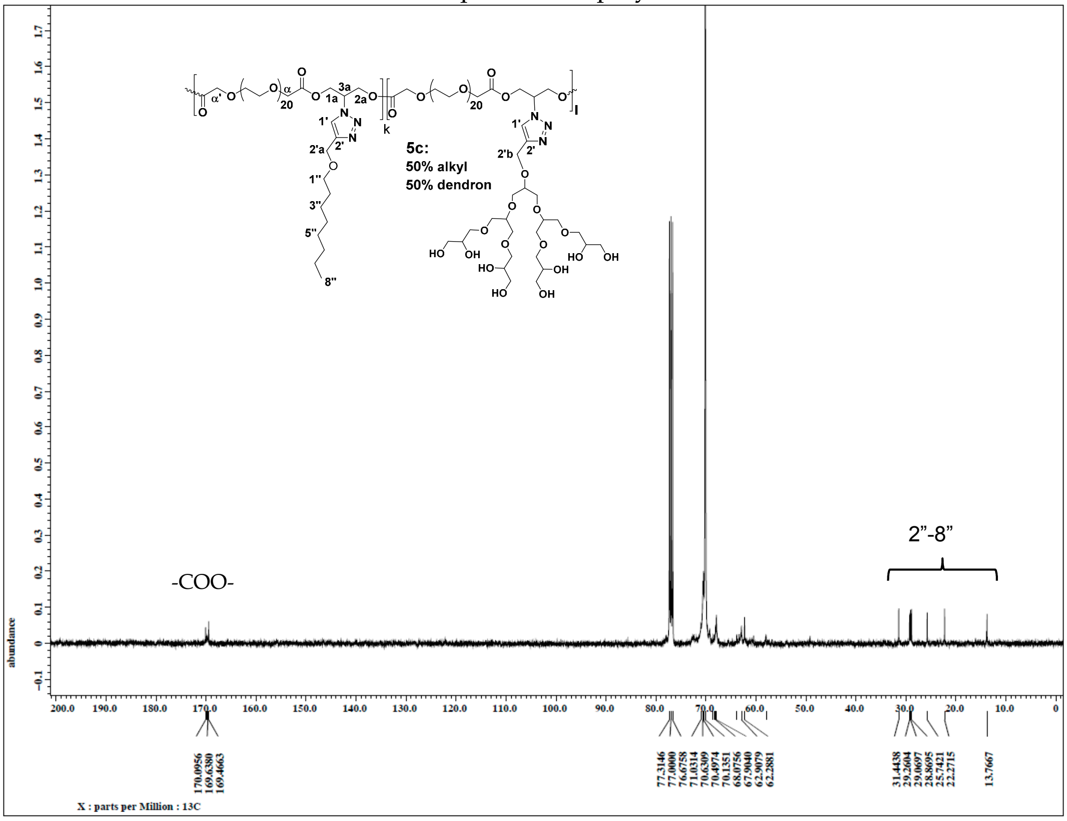

^{13}C NMR spectrum of polymer 5c

Figure S4. ^{1}H and ^{13}C NMR spectra of polymer **5c**.

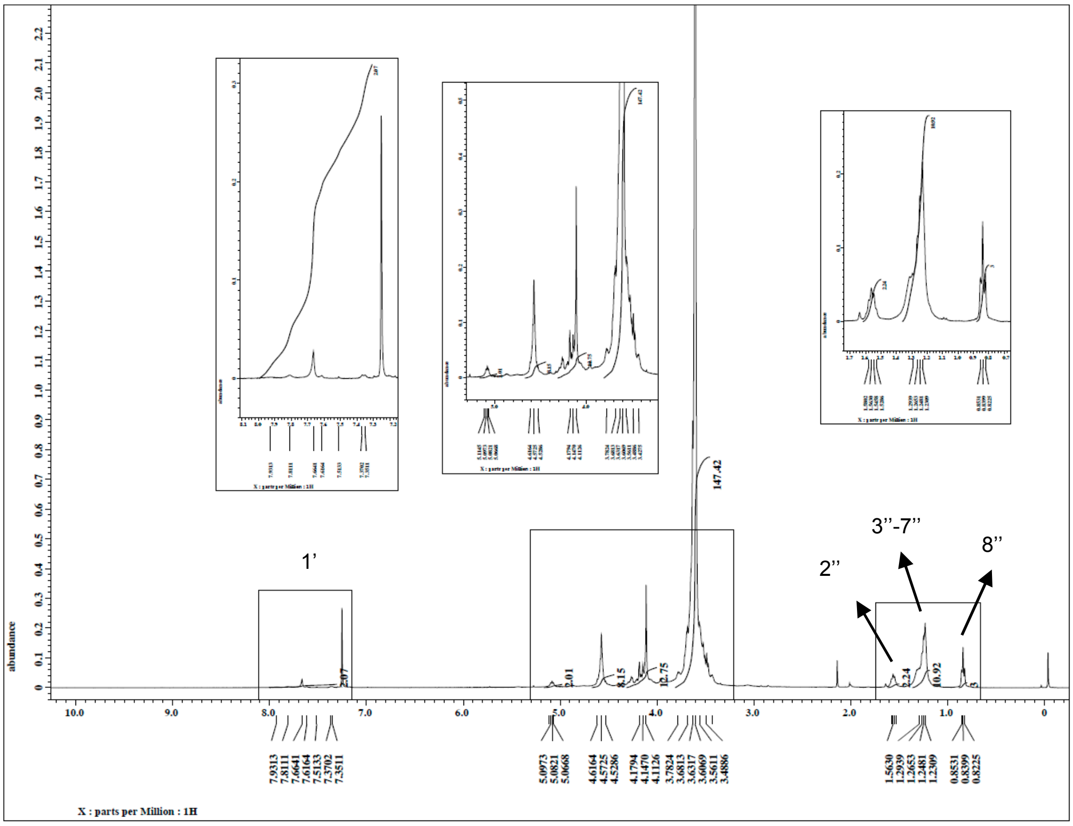

¹H NMR spectrum of polymer 5d

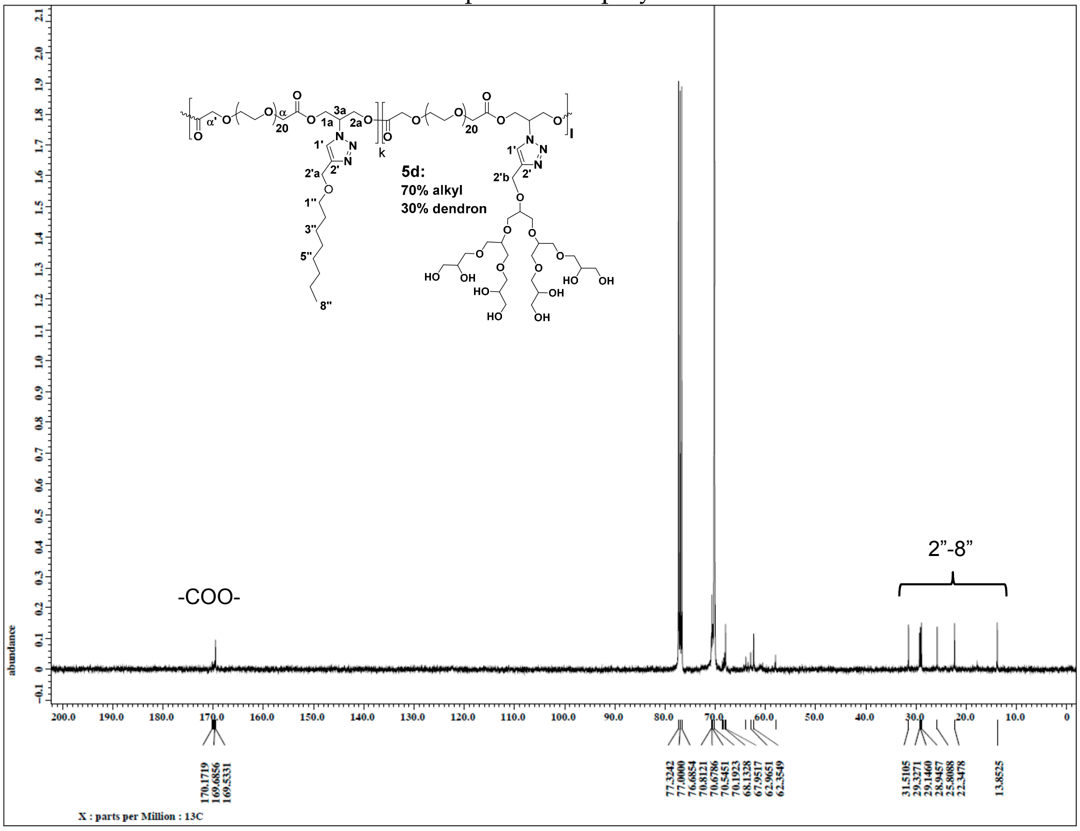

¹³C NMR spectrum of polymer 5d

Figure S5. ¹H and ¹³C NMR spectra of polymer **5d**.

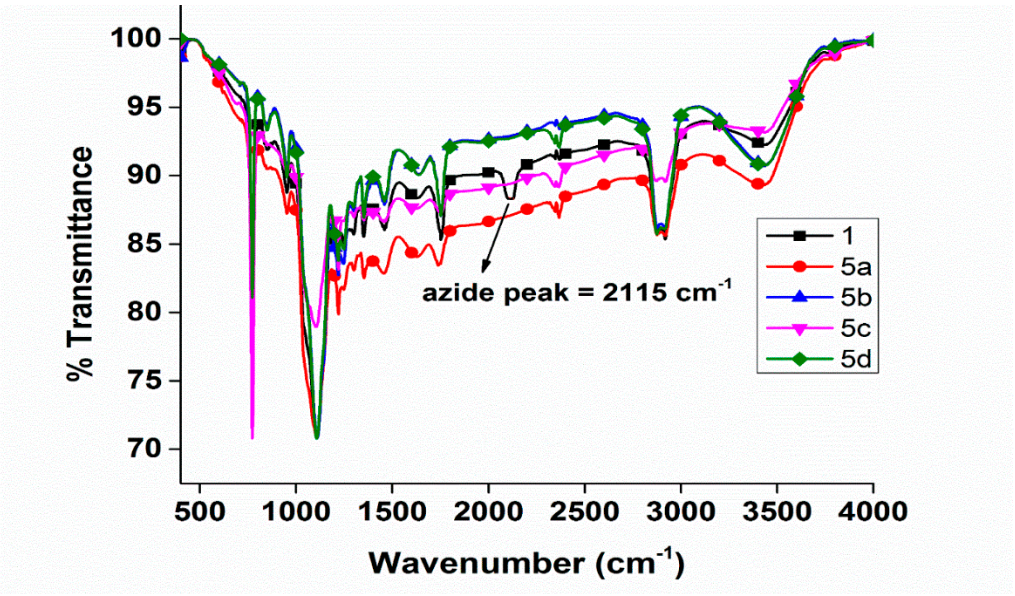

Figure S6. IR spectra of polymers (**1** and **5a–5d**).

Gel permeation chromatogram; Detector: RI, Eluent: THF, Flow rate: 1 mL/min, Standard: Pullulan

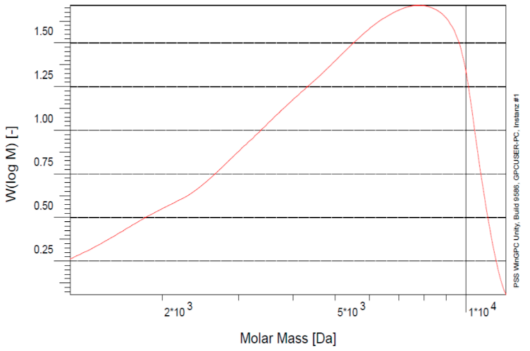

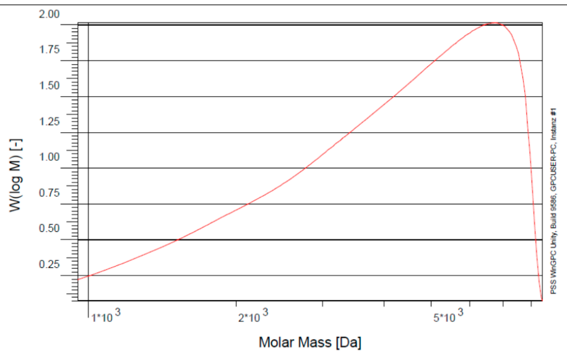

(A) Polymer 5a

$\overline{M}_w$ = 5552 g/mol, $\overline{M}_n$ = 4127 g/mol, $\overline{M}_z$ = 6811 g/mol, D = 1.3

(B) Polymer 5b

$\overline{M}_w$ = 4336 g/mol, $\overline{M}_n$ = 3284 g/mol, $\overline{M}_z$ = 5210 g/mol, D = 1.3

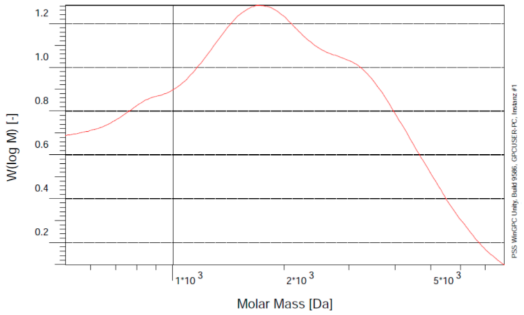

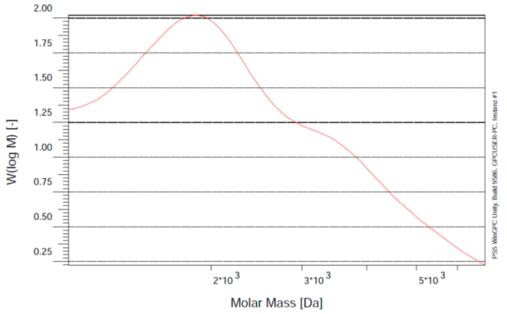

(C) Polymer 5c

$\overline{M}_w$ = 2162 g/mol, $\overline{M}_n$ = 1431 g/mol, $\overline{M}_z$ = 3109 g/mol, D = 1.5

(D) Polymer 5d

$\overline{M}_w$ = 2454 g/mol, $\overline{M}_n$ = 2011 g/mol, $\overline{M}_z$ = 3046 g/mol, D = 1.2

Figure S7. GPC chromatogram of polymers **5a–5d**.

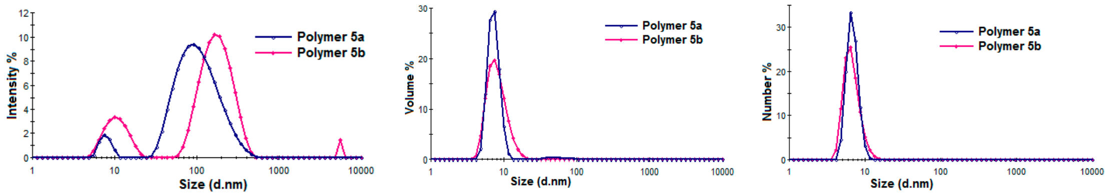

Figure S8. DLS size distribution graphs of polymers**5a** and **5b**.

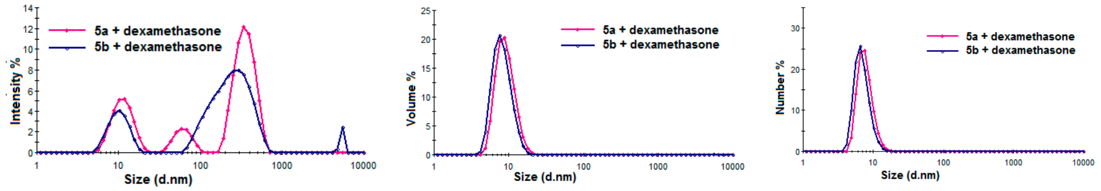

Figure S9. DLS size distribution graphs of dexamethasone encapsulated polymers **5a** and **5b**.

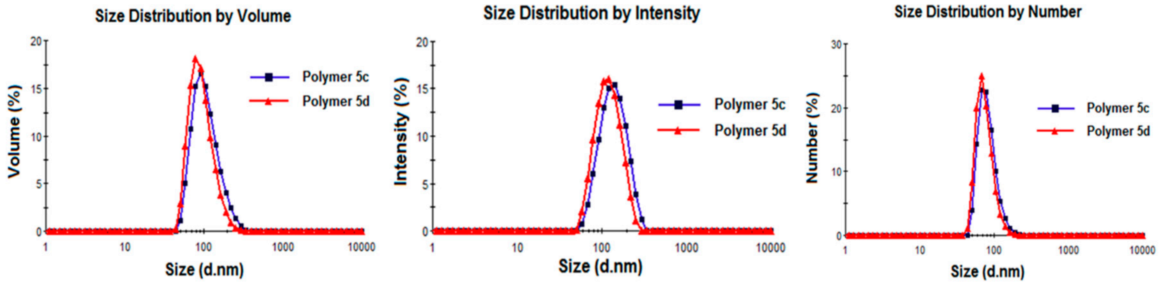

Figure S10. DLS size distribution graphs of polymers**5c** and **5d**.

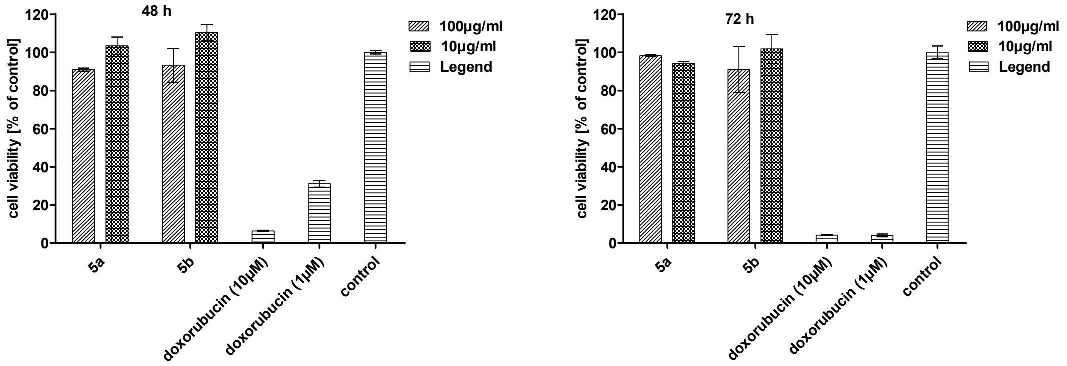

Figure S11. Cytotoxicity study of the polymers **5a** and **5b** at concentration of 10 and 100 μg/mL for 48 and 72 h using HeLa cells.

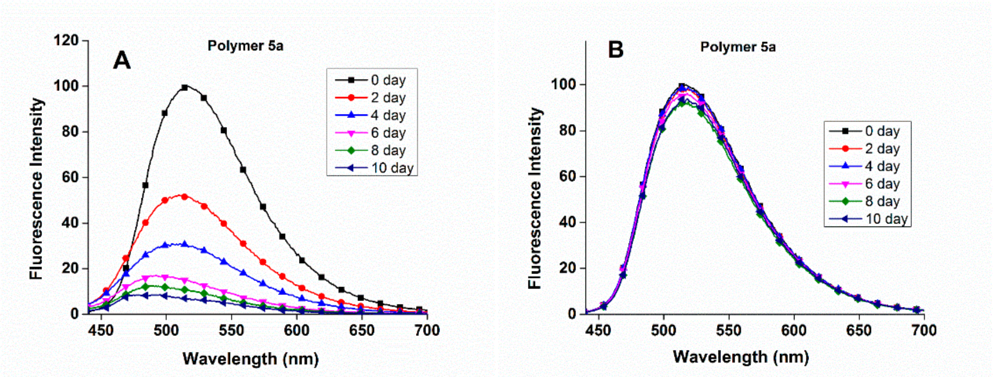

Curcumin release from polymer **5a**, (A) In presence of enzyme; (B) In absence of enzyme

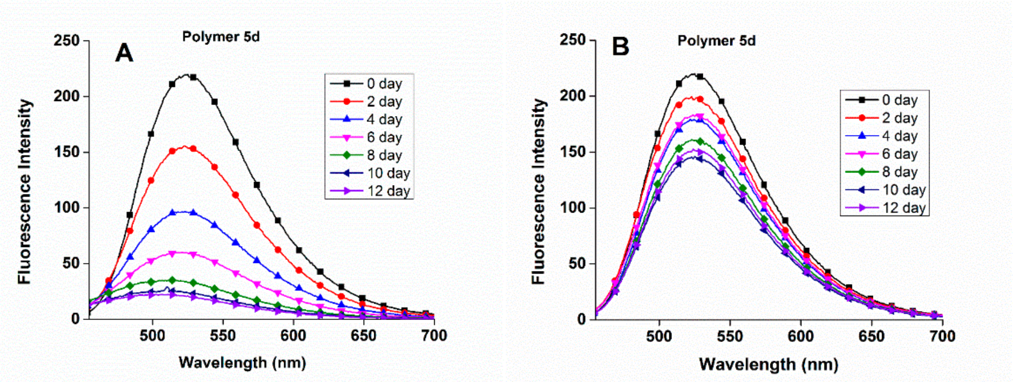

Curcumin release from polymer **5d**, (**A**) In presence of enzyme; (**B**) In absence of enzyme

Figure S12. Fluorescence measurement of curcumin's release from polymers **5a** and **5d**, with and without presence of enzyme.

For polymer **5a**

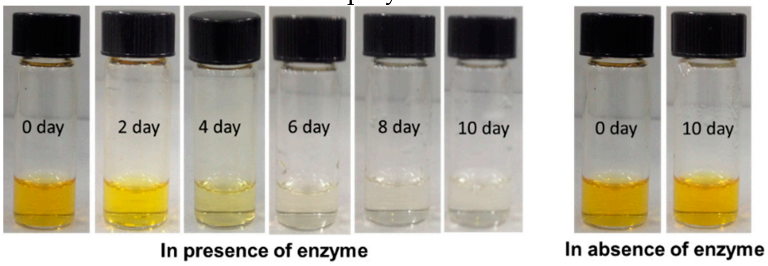

For Polymer **5d**

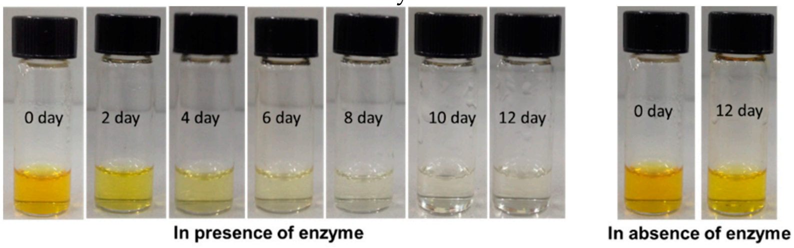

Figure S13. Time dependent release of curcumin from polymers **5a** and **5d**, with/without incubation of enzyme.

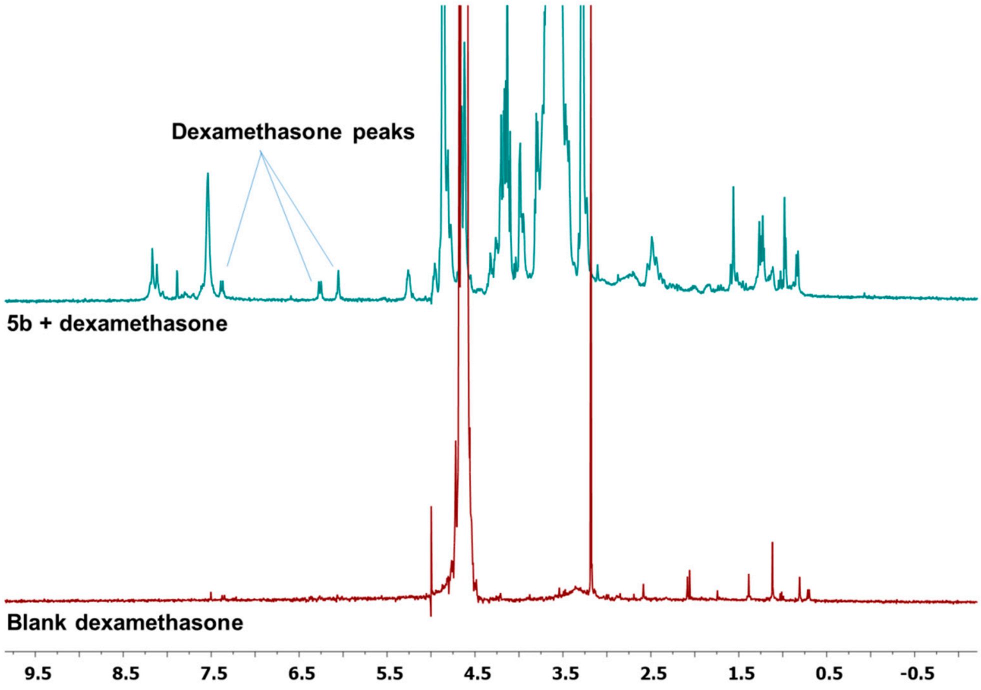

Figure S14. ^{1}H NMR spectra of dexamethasone (Blank), and **5b** + dexamethasone in D_2O.

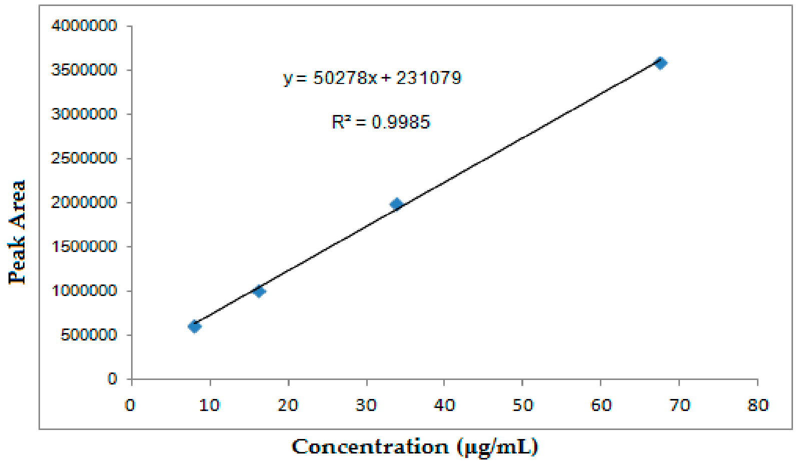

Figure S15. Calibration graph of dexamethasone; Peak Area: Y-axis and Concentration of dexamethasone: X-axis (in µg/mL).

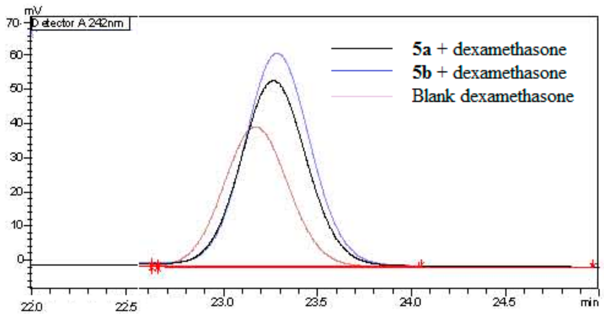

Figure S16. HPLC chromatogram of dexamethasone encapsulated in fluorinated polymeric samples using acetonitrile:water:phosphoric acid (30:70:0.5, *v*/*v*/*v*) as an eluent.

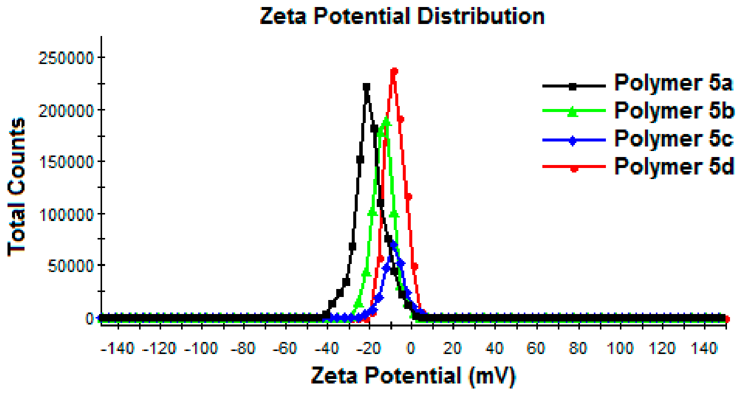

Figure S17. Mean Zeta Potential of Polymers **5a–5d**.

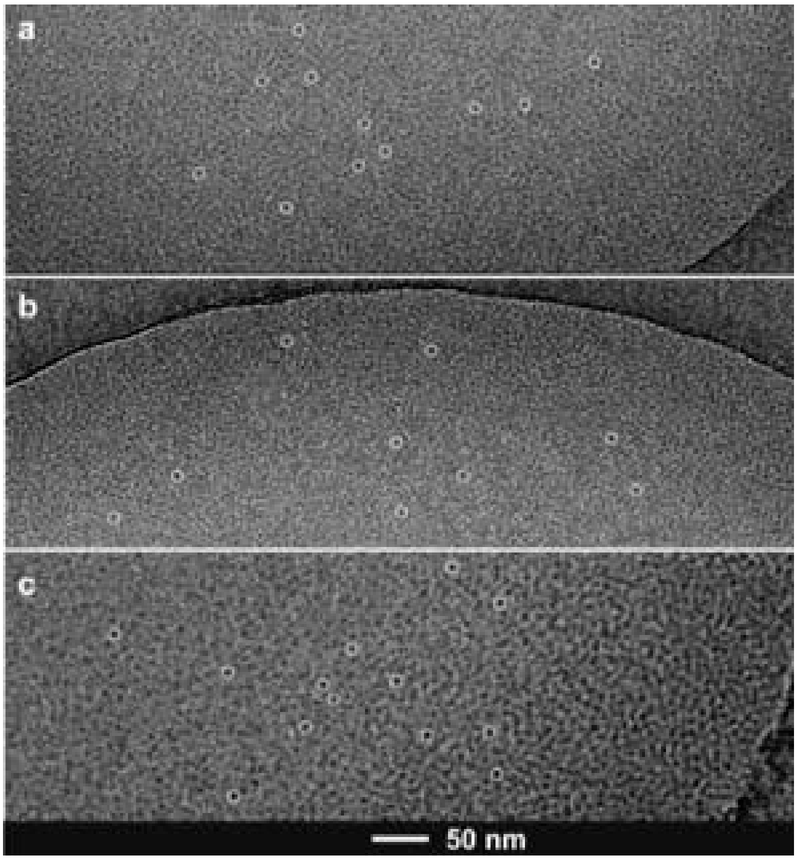

Figure S18. Cryo-TEM images of polymers, (a) **5a**; (b) **5b** (c) curcumin encapsulated polymer **5a**.

Table S1. Transport behaviour, Encapsulation efficiency and Zeta Potential of Polymers **5a**–**5d**.

Polymer	Transport behavior (Curcumin/Polymer)		Curcumin Encapsulation efficiency (%)	Dexamethasone Encapsulation efficiency (%)	Zeta Potential (mV)
	mg/g	mmol/mol			
5a	5.34	213.17	1.6	2.12	−19.7
5b	4.52	176.75	1.4	2.48	−14.0
5c	2.67	98.95	0.8	-	−8.9
5d	3.86	135.41	1.2	-	−7.9

© 2016 by the authors; licensee MDPI, Basel, Switzerland. This article is an open access article distributed under the terms and conditions of the Creative Commons Attribution (CC-BY) license (http://creativecommons.org/licenses/by/4.0/).
